# Retraction: The Impact of Intraoperative Imaging on Outcomes in Combined Neurosurgical and Reconstructive Procedures: A Systematic Review

**DOI:** 10.7759/cureus.r195

**Published:** 2025-10-17

**Authors:** Muhammad Ilyas Alozai, Omar Amgad Yehia Elassra, Aliaa H Alkhazendar, Ahmed S Ibrahim, Abdul Sattar Gatta, Syed Muhammad Baqar Raza, Abdelrahman Sahnon Abaker Sahnon, Jarallah HJ Alkhazendar, David O Oriko, Shafaq Mushtaq

**Affiliations:** 1 Internal Medicine, Punjab Medical College, Faisalabad Medical University, Faisalabad, PAK; 2 Emergency Medicine, North Manchester General Hospital, Manchester, GBR; 3 Plastic Surgery, Ain Shams University, Cairo, EGY; 4 Surgery, The Islamic University of Gaza, Gaza, PSE; 5 Orthopedics and Traumatology, Royal Care International Hospital, Khartoum, SDN; 6 Accident and Emergency, Liaquat National Hospital, Karachi, PAK; 7 Medicine, Liaquat College of Medicine and Dentistry, Jinnah Sindh Medical University, Karachi, PAK; 8 Cardiac Surgery, Rawalpindi Institute of Cardiology, Rawalpindi, PAK; 9 Vascular Surgery, Evangelisches Krankenhaus Hubertus, Berlin, DEU; 10 General Physician, Speedy Recovery Clinic, Jeddah, SAU; 11 General and Emergency Surgery, East and North Hertfordshire NHS Trust - Lister Hospital, Stevenage, GBR; 12 Neurology, University of Maryland Medical Center, Baltimore, USA; 13 Surgery, Liaquat National Hospital, Karachi, PAK

The Editors-in-Chief have retracted this article. An investigation by the journal found evidence of authorship manipulation. The Editors-in-Chief therefore no longer have confidence in the provenance and reliability of the article contents. The authors disagree with the decision to retract.

